# Single or Vortex Ferroelectric and Ferromagnetic Domain Nanodot Array of Magnetoelectric BiFe_0.9_Co_0.1_O_3_

**DOI:** 10.1021/acsami.4c01232

**Published:** 2024-04-09

**Authors:** Keita Ozawa, Yasuhito Nagase, Marin Katsumata, Kei Shigematsu, Masaki Azuma

**Affiliations:** † Laboratory for Materials and Structures, Institute of Innovative Research, Tokyo Institute of Technology, Nagatsuta, Midori-ku, Yokohama, Kanagawa 226-8501, Japan; ‡ Kanagawa Institute of Industrial Science and Technology (KISTEC), Shimoimaizumi, Ebina, Kanagawa 243-0435, Japan; § Sumitomo Chemical Next-Generation Eco-Friendly Devices Collaborative Research Cluster, Tokyo Institute of Technology, Yokohama 226-8501, Japan; ∥ Living Systems Materialogy (LiSM) Research Group, International Research Frontiers Initiative (IRFI), Tokyo Institute of Technology, Yokohama 226-8501, Japan

**Keywords:** multiferroics, bismuth ferrite, nanodot, topological domain, anodized alumina

## Abstract

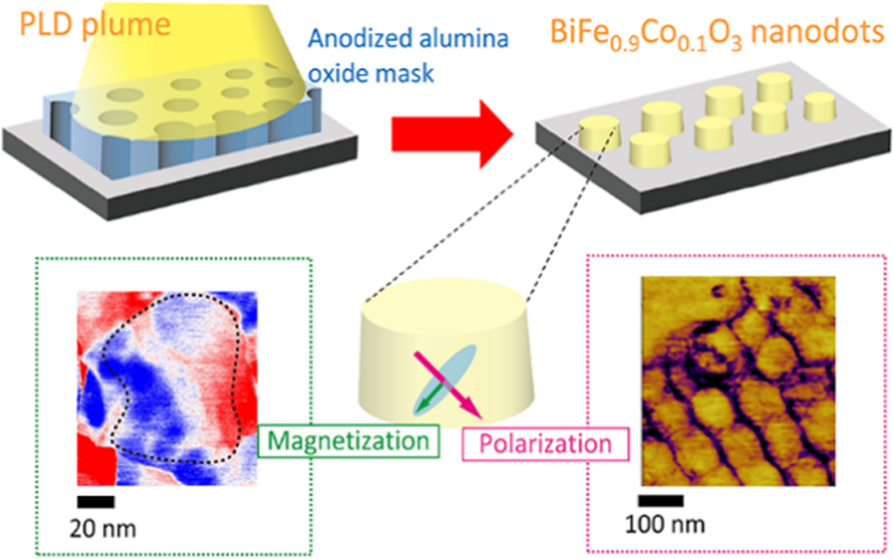

Nanodots composed of multiferroic cobalt-substituted BiFeO_3_, a ferroelectric ferromagnet at room temperature, are fabricated by pulsed laser deposition using anodized porous alumina as masks. The obtained nanodots are approximately 60 nm in diameter, more than 10 nm in thickness, and approximately 70 Gbit/in.^2^ in density. Piezoresponse and magnetic force microscopies show both ferroelectricity and ferromagnetism with a single-domain nature. It is also found that the dot with 190 nm diameter had multidomain vortex ferroelectric and magnetic structures indicating the strong magnetoelectric coupling. The single-domain cobalt-substituted BiFeO_3_ nanodots are suitable for verifying magnetization reversal by the electric field, which is the first step in the development of low-power-consumption nonvolatile magnetic memory devices.

## 1 Introduction

Multiferroic materials exhibiting ferroelectric and ferromagnetic orders have possible applications in ultralow-power-consuming memory devices. In particular, if the magnetization can be reversed by an electric field, an electric-field writing magnetic read-out memory device can be fabricated. Such a memory device is energetically advantageous as a recording medium because it would avoid the energy-consuming writing process of magnetic memories and the destructive read-out process of ferroelectric memories.^1−6^

**Figure 1:**
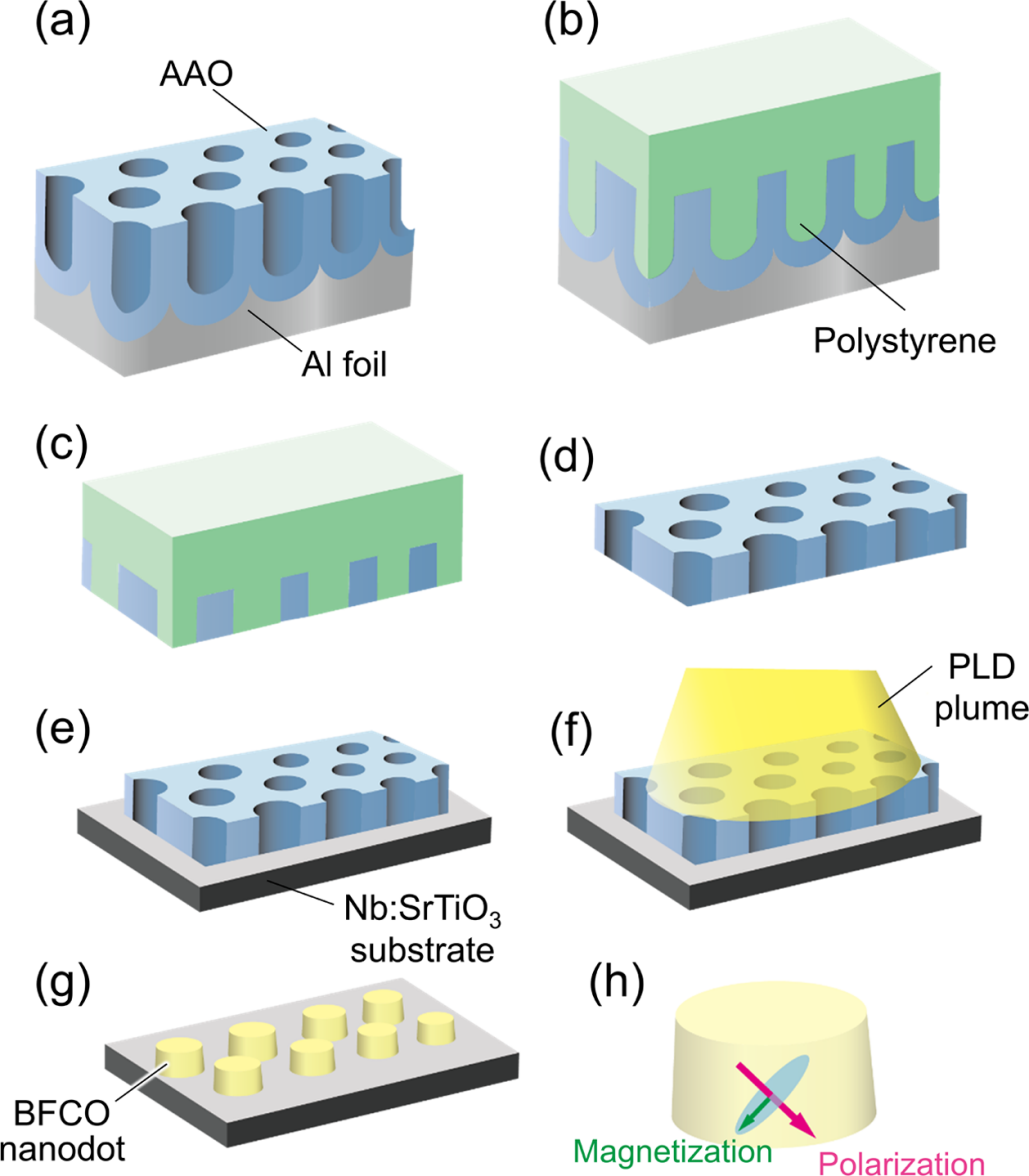
Scheme for fabricating BFCO nanodots on Nb:STO substrate: (a) AAO formed on Al foil, (b) coating polystyrene as a protection layer, (c) dissolving Al foil and removing the bottom of AAO, (d) transferring the AAO mask to Nb:SrTiO_3_ substrate, (e) removing the protection layer, (f) growing BFCO nanodots by PLD, and (g) removing the AAO mask. (h) Schematic illustration of a ⟨111⟩_pc_ polarization and magnetic easy-plane perpendicular to the polarization in a single BFCO nanodot.

Perovskite bismuth ferrite (BiFeO_3_; BFO, space group: *R*3*c*) is the most widely studied multiferroic material. It has robust ferroelectricity with a large electric polarization ∼90 μC cm^–2^ and G-type antiferromagnetism of spin 5/2 of Fe^3+^ ions at room temperature.^7,8^ A net magnetization is necessary for magnetic memory application, but while a net magnetization owing to spin canting is theoretically predicted, the presence of cycloidal modulation with a period of 62 nm propagating in the hexagonal [110] direction prohibits its appearance.^9^ We have shown that partial substitution of Co^3+^ for Fe^3+^ in BiFe_1–*x*_Co_*x*_O_3_ (*x* ≤ 0.2) stabilizes the collinear spin structure with weak ferromagnetism preserving the polar *R*3*c* crystal structure.^10−13^ Spontaneous magnetization is therefore generated close to perpendicular to the electric polarization due to the Dzyaloshinskii–Moriya interaction, as schematically illustrated in Figure 1h,^14^ unless the deviation of spin direction due to lattice strain from the substrate^15,16^ is significant. Moreover, we have demonstrated magnetization reversal by the electric field in BiFe_0.9_Co_0.1_O_3_ (BFCO) thin films on a GdScO_3_ (GSO) (110)_o_ (o denotes orthorhombic) substrate by employing a combination of piezoresponse force microscopy (PFM) and magnetic force microscopy (MFM). PFM and MFM images before and after application of an electric field perpendicular to the thin film were compared, and an out-of-plane magnetization reversal from 71° polarization switching without ferroelectric domain reconstruction was confirmed.^17,18^ This was the first direct observation of magnetization reversal by an electric field at room temperature.

The next step in the development of a multiferroic memory device is the fabrication of nanodots with the size of a single ferroelectric and ferromagnetic domain. BFO nanodots were reported to be obtained by bottom-up methods (mask-assisted deposition or self-assembly) and top-down methods (dry etching or ion milling).^19−27^ In particular, nanodots have been formed by dry etching with Ar^+^ ions, and various ferroelectric domain structures such as vortex domain, multidomain, or single domain have been reported.^21−24^ However, it is not obvious that a dot with a single ferroelectric domain structure has a magnetic single domain. Moreover, the magnetic domain structure with magnetoelectric multiferroic materials with a vortex ferroelectric domain has never been investigated. However, the Ar^+^ ion bombardment may lead to the production of magnetic impurities (i.e., γ-Fe_2_O_3_) in BFCO, which hampers observation of the intrinsic magnetic signals.

In this study, we utilized regularly aligned nanometer-scale pores in anode alumina oxide (AAO)^28−31^ as a template for the nanodot fabrication and clarified that a dot with 60 nm has single ferroelectric and ferromagnetic domain while that with 190 nm has multidomain structures. We deposited BFCO nanodots on a conductive Nb:SrTiO_3_(001) substrate by pulsed laser deposition (PLD) with AAO as a mask (Figure 1a–f). This synthesis route has the following advantages: (i) formation of magnetic impurities during the etching process can be avoided; (ii) AAO is tolerant to the high temperature needed for the epitaxial growth of BFCO; (iii) the pore size of AAO is 10–400 nm in diameter and is tunable by varying the anodization conditions,^32^ which enables the ferroelectric/magnetic domains of BFCO nanodots to be controlled by the size effect. The ferroelectric and weakly ferromagnetic natures of the film fabricated in the same PLD condition as the dots are confirmed by PFM and magnetization measurements as shown in Figure S1a,b. The magnetoelectric coupling of BFCO nanodots with the two different sizes is investigated by a comparison of ferroelectric and magnetic domain structures.

## 2 Results and Discussion

**Figure 2:**
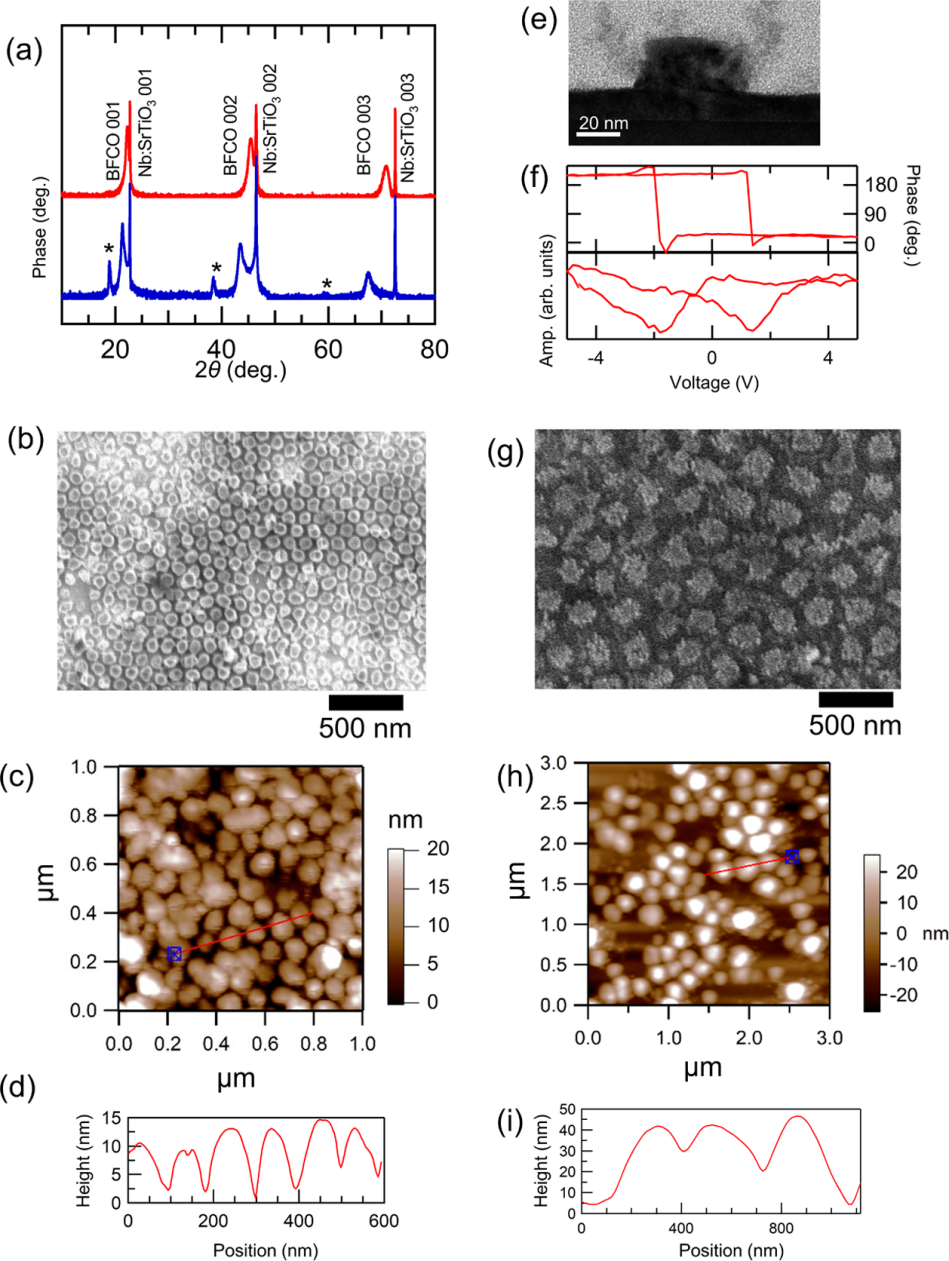
(a) XRD 2θ–ω pattern from BFCO obtained with oxalic acid (blue) and malonic acid (red) AAO masks. Asterisks indicate the giant tetragonal BFCO phase. (b) FE-SEM image of oxalic acid AAO nanodot array. (c, d) AFM image of BFCO oxalic acid AAO array and the height profile along the red line. (e) Cross-sectional TEM image of BFCO oxalic acid nanodot. (f) Hysteresis loop of PFM phase (top) and amplitude (bottom) obtained by sweeping the tip bias on a single BFCO oxalic acid AAO nanodot. (g) FE-SEM image of BFCO malonic acid AAO nanodot array. (h, i) AFM image of BFCO malonic acid AAO nanodot array and the height profile along the red line.

Figure 2a shows the out-of-plane 2θ–ω XRD patterns of BFCO nanodots grown on Nb:STO(001) substrates through AAO masks fabricated by anodization in oxalic acid (oxalic acid AAO nanodots) and malonic acid (malonic acid AAO nanodots). It should be noted that since the coverage of the AAO mask on the substrate was not 100%, the XRD signal included both the nanodot area and the thin-film area outside the AAO mask (see Figure S3b). The 00*l* peaks next to the substrate peaks were close to monoclinic phase of BFCO,^33,34^ confirming the epitaxy of the nanodots. The out-of-plane lattice constants were 4.12 and 3.99 Å for BFCO oxalic acid AAO nanodots and malonic acid AAO nanodots, respectively. This may be attributed to the difference in oxygen vacancies in BFCO depending on the growth conditions during the PLD process. The XRD of BFCO oxalic acid AAO nanodots shows diffractions from a secondary phase with a large out-of-plane lattice spacing of ∼4.62 Å (marked by asterisks). This value is close to that of a thin film of the giant tetragonal BFO on SrTiO_3_.^35^ Giant tetragonal BFO is not lattice-matched to SrTiO_3_, but at a high deposition rate, the epitaxial growth is possible by the formation of the β-Bi_2_O_3_ phase at the interface, which can act as a buffer layer.^36^ In our PLD process, the nanodots form through the holes in the AAO mask, so the deposition rate of the nanodots is much smaller than that of the thin-film area. Therefore, it is reasonable to assume that the giant tetragonal phase is present only in the thin-film area with high deposition late and not in the nanodot area. Other impurities that might affect the properties of BFCO nanodots, such as Fe_2_O_3_, were not detected by XRD.

The SEM image of oxalic acid AAO BFCO nanodots (Figure 2b) confirms the presence of regularly aligned nanodots. The average diameter and interdot distance were evaluated to be approximately 60 and 100 nm, respectively. These values correspond to a dot density of ∼70 Gbit/in.^2^. The spatial distribution and diameter of the nanodots correspond to those of the porous array of the AAO mask. The topographic image acquired by AFM is shown in Figure 2c. The line profile (Figure 2d) along the red line suggests that the height of the nanodot was more than 10 nm. These topographic results were consistent with the observations from the cross-sectional transmission electron microscopy (TEM) image (Figure 2e). The oxalic acid AAO BFCO nanodots had a sufficiently small diameter satisfying the conditions for a single-domain structure that were estimated from the previous studies (<100 nm). On the other hand, malonic acid AAO nanodots had the average diameter of 190 nm and height of 30 nm as indicated by SEM and AFM images shown in Figure 2g–i. To verify the ferroelectricity of the BFCO nanodots, the PFM phase and amplitude were measured in a single dot by sweeping the voltage via a cantilever. Figure 2f displays a typical result on a 60 nm dot (oxalic acid AAO BFCO nanodot), showing clear hysteresis and butterfly-shaped loops in the phase and amplitude plots. The hysteresis shift to the negative voltage side would be attributed to several reasons including the pinning of the downward polarization due to the lattice strain, strain gradient, defects, or asymmetric film–electrode interfaces.

**Figure 3:**
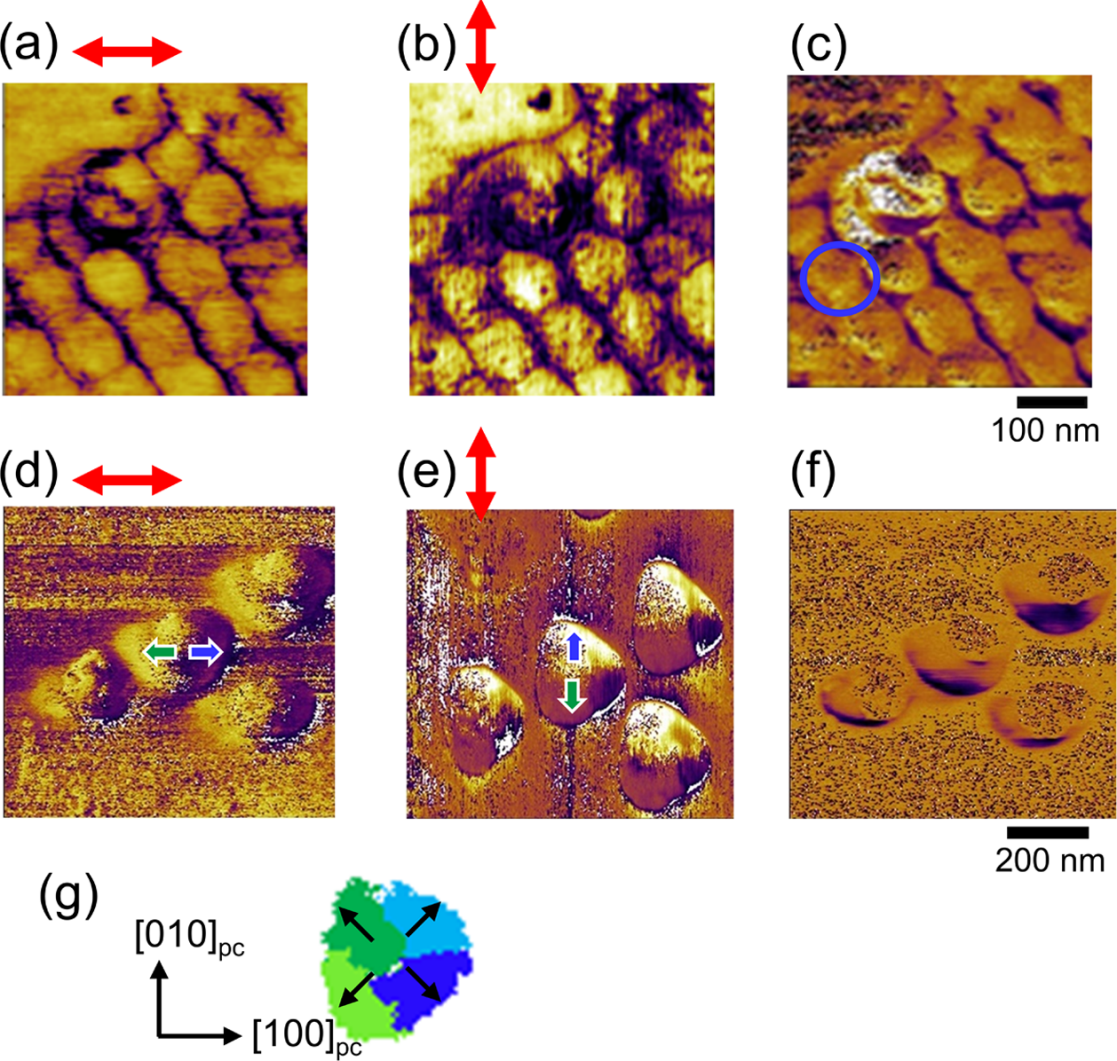
PFM phase images on BFCO nanodot arrays. (a, b) Lateral PFM phases (the red arrows indicate the identifying direction) of the oxalic acid AAO nanodot array. (c) Vertical PFM phase image of the oxalic acid AAO nanodot array. (d, e) Lateral PFM phases (the red arrows indicate the identifying direction) of the maloic acid AAO nanodot array. (f) Vertical PFM phase image of the malonic acid AAO nanodot array. (g) Schematic of ferroelectric domain distribution inside a single nanodot.

Next, we observed the polarization and magnetic domain structures in these samples. Figure 3a–c depicts lateral and vertical PFM phase images of oxalic acid AAO nanodots. Most nanodots have single contrast without domain boundaries, indicating formation of single ferroelectric domains. On the other hand, domain boundaries were observed in both in-plane directions (Figure 3d,e) in the malonic acid AAO nanodots with an average diameter of 190 nm, though the out-of-plane PFM contrast was uniform (Figure 3f). This indicates a generation of vortex domains, as schematically visualized in Figure 3g, because the lateral size of nanodots became larger than the width of ferroelectric stripe-domains of BFCO thin films (∼100 nm).^17^ Such a variation of ferroelectric domain structures as a function of the nanodot diameter agrees with the BiFeO_3_ nanodots, where the ferroelectric domain changed as stripe-domain structures with 71° boundaries at 900 nm in diameter, vortex domains at ∼400 nm, and single domains at 100 nm.^24^ We also attempted to make out-of-plane polarization switching with a biased cantilever of −8 V (Figure S4). As a result, the contrast change can be confirmed at the poled region, but unfortunately, the poled state was preserved only for 10 min, most probably because of the insufficient height of the dots.

**Figure 4:**
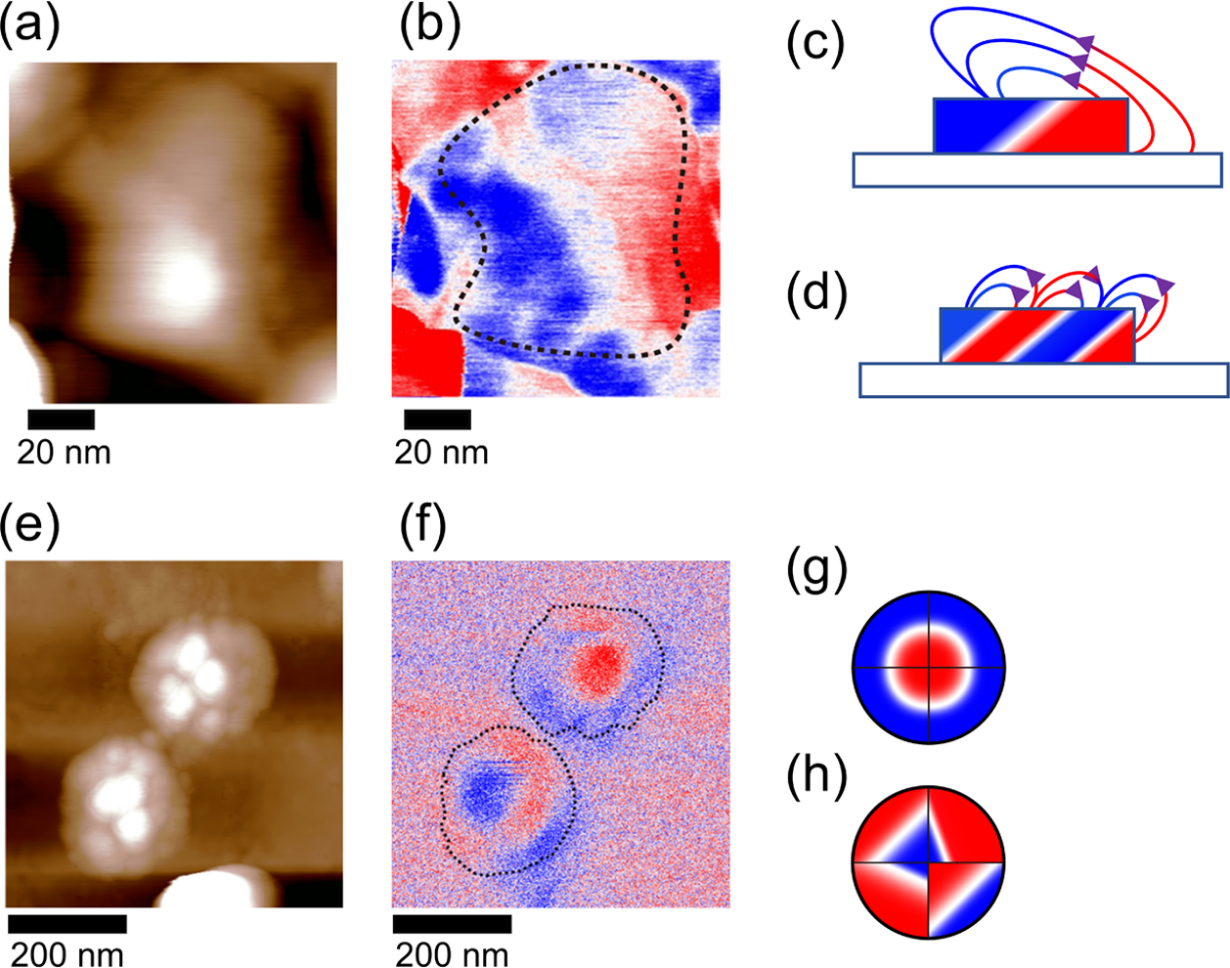
MFM observations of single BFCO nanodots. (a) AFM topographic image of an oxalic acid AAO nanodot marked with the blue circle in Figure 3c measured with an SSS-MFMR cantilever. (b) MFM image. The dashed line indicates the outward form of the dots. (c, d) Schematic illustrations of expected magnetic flux and contrast in MFM image for comparing magnetic monodomain and multiple domains. (e, f) AFM topographic and MFM image of oxalic acid AAO nanodots. The dashed line indicates the outward form of the dots. (g, h) Schematic MFM contrast explaining the magnetoelectic coupling in the topological vortex domain.

Finally, we observed the ferromagnetic domain by MFM. Figure 4a,b depicts the topology and MFM phase images of oxalic acid AAO nanodot, respectively. The dashed line in panel (b) indicates the outward form of the dot. The red-blue contrast in the MFM image represents the upward or downward magnetic fields leaking from the nanodot sensed by the magnetic cantilever. The nanodot has a blue contrast at one end (left) and a red contrast at the opposite end (right), with a straight domain boundary between them. Note that these MFM measurements were conducted with both upward and downward tip magnetizations to confirm the contrast in MFM images truly comes from magnetic interaction (Figure S5). Given that BFCO has a magnetic easy-plane perpendicular to the electric polarization inclined from the surface as shown in Figure 1h, we suppose that the red and blue in the MFM image illustrated in Figure 4c represent the magnet poles. That is, this nanodot has a single magnetic domain. If the nanodot was multidomain, its magnetic domain would look like the schematic image in Figure 4d, and multiple-domain boundaries would be observed in a single nanodot.

The MFM image of malonic acid AAO nanodots (Figure 4f) shows multiple-domain boundaries. However, two nanodots in this MFM image look very different though their ferroelectric domains have similar vortex structures as shown in Figure 3g. Therefore, at first glance, there seems to be no correlation between the ferromagnetic and ferroelectric domains each other. However, the structure of the magnetic domain can be explained by extending the result for the oxalic AAO acid nanodots, as described below. Here, we simplify the vortex domain structure to four domain variants. Then, as is similar to the case for oxalic acid AAO nanodots, we assume that there are red and blue regions corresponding to a single magnetic pole per domain variant. Based on this consideration, the upper nanodot in Figure 4f can be understood as all domain variants have a magnetic pole with the red part near the center and the blue part near the edge, as schematically shown in Figure 4g. As for the bottom nanodots, the MFM contrast changed from blue, red, to blue along the upper left to lower right. This can be schematically drawn in Figure 4h, where one of the ferroelectric variants has a reversed magnetic pole to the others. Since the magnetic pole lies inside the ferroelectric domains, their domain boundaries in PFM and MFM images are not coincident, which is common for both sizes of nanodots. This feature is distinct from the observation in BFCO thin films exhibiting similar striped PFM and MFM domain structures with the same location of domain boundaries.^17^ We thus believe the observed magnetic domains in BFCO nanodots are characteristic of vortex domains, indicating the magnetoelectric coupling.

Such single- or multiple-domain structures of ferroelectricity and ferromagnetism would be an ideal platform for investigating BFCO as an electric-field writing magnetic read-out memory device. The investigation of nanodots with additional different sizes is expected to provide more useful information on ferroelectric and ferromagnetic domain structures of BFCO, but unfortunately, the hole sizes in the AAO mask are limited by the acid solution for the anodization process and PLD conditions for the growth of BFCO. The application of alternative techniques such as electron beam lithography would be promising. It would also be interesting to investigate how the properties of ferroelectricity and ferromagnetism of BFCO nanodots change when their size is further reduced, as this would be an important prospect for memory applications.

## 3 Conclusions

BFCO nanodots were fabricated by employing the AAO-assisted PLD method. The obtained nanodots were ∼60 and ∼190 nm in diameter and more than 10 nm in thickness, and the former dot density corresponded to ∼70 Gbit/in.^2^. Furthermore, PFM and MFM observations confirmed the single-domain nature of the ferroelectric and ferromagnetic domains for 60 nm nanodots while both ferroelectric and ferromagnetic domains were multidomains in 190 nm nanodots. Strong magnetoelectric coupling is indicated by the comparison of ferroelectric and magnetic domain structures. BFCO 60 nm nanodots are promising for high-density, nonvolatile magnetic memory devices with ultralow power consumption.

## 4 Experimental Methods

The AAO was used as a mask for the PLD synthesis of BFCO nanodots. It was prepared by two-step anodization of Al foil in oxalic acid or malonic acid followed by dissolution of the Al metal, as reported in the literature.^37^ The pores’ structure was checked with a field-emission scanning probe microscope (FE-SEM: Hitachi High-Tech S-4800). The AAO was transferred to the Nb:SrTiO_3_(001) substrate. Regarding the growth of the BFCO nanodots by PLD, the substrate temperature and KrF laser fluence were 535 °C and 1.0 J/cm^2^ for oxalic acid AAO nanodots and 560 °C and 0.8 J/cm^2^ for malonic acid AAO nanodots, respectively. The oxygen partial pressure, repetition rate of the pulse laser, and deposition time were set to 1.4 Pa, 2 Hz, and 2.5 h, respectively. Note that the oxygen partial pressure was lower than in our previous study^38^ in order to increase the amount of ablated species through the AAO mask. After the PLD process, the AAO was mechanically eliminated with the aid of sodium hydroxide.

Topographic images of BFCO nanodots were obtained by atomic force microscopy (AFM: Cypher S was obtained from Asylum Research) and by FE-SEM. The crystal structure was examined with an X-ray diffractometer (XRD: Rigaku SmartLab). The ferroelectric and magnetic domains were observed by PFM and MFM with the Cypher S. The measurement conditions were the same as in our previous paper,^17^ but we changed the magnetically coated tip to a sharper one (Nanosensors SSS-MFMR: magnetic resolution <25 nm) to obtain high-resolution MFM images.

## Supporting Information Available

PFM image and *M*–*H* curve of the BFCO film prepared in the same condition as nanodots, SEM images of AAO mask, PFM image of poled BFCO nanodots, and reversal of MFM contrast by changing the magnetization direction of MFM tip (PDF)
