# Supplementary material for: Single or Vortex Ferroelectric and Ferromagnetic Domain Nanodot Array of Magnetoelectric BiFe0.9Co0.1O3
Source: ACS Appl Mater Interfaces. 2024 Apr 9;16(16):20930–6. doi: 10.1021/acsami.4c01232 (PMC11056924; doi:10.1021/acsami.4c01232)
Supplement: Supplementary file 1 — am4c01232_si_001.pdf [file am4c01232_si_001.pdf]

Supporting Information

Single or vortex ferroelectric and ferromagnetic domain nanodot array of magnetoelectric $BiFe_{0.9}Co_{0.1}O_3$

Keita Ozawa[1], Yasuhito Nagase[1], Marin Katsumata[1], Kei Shigematsu[1,2,3]*, and Masaki Azuma[1,2,3,4]

[1]Laboratory for Materials and Structures, Institute of Innovative Research, Tokyo Institute of Technology, Nagatsuta, Midori-ku, Yokohama, Kanagawa 226-8501, Japan

[2]Kanagawa Institute of Industrial Science and Technology (KISTEC), Shimoimaizumi, Ebina, Kanagawa 243-0435, Japan

[3]Sumitomo Chemical Next-Generation Eco-Friendly Devices Collaborative Research Cluster, Tokyo Institute of Technology, Yokohama 226-8501, Japan

[4]Living Systems Materialogy (LiSM) Research Group, International Research Frontiers Initiative (IRFI), Tokyo Institute of Technology, Yokohama 226-8501, Japan

Email: kshigematsu@msl.titech.ac.jp

PFM image and M-H curve of the BFCO film prepared in the same condition as nanodots

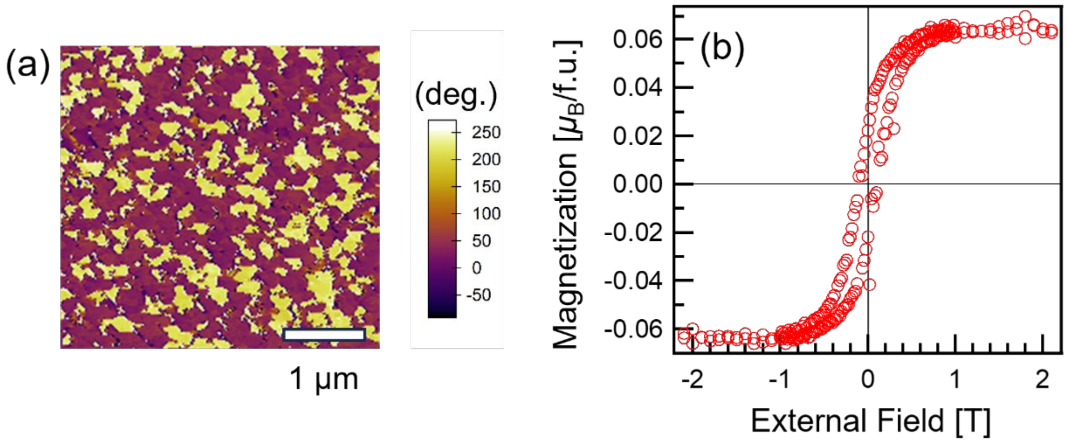

Figure S1. (a) In-plane PFM phase image and (b) *M-H* hysteresis loop at 300 K for the films produced by PLD in the same condition used in the nanodot process in this work.

SEM image of AAO mask anodized with oxalic acid and malonic acid

The SEM images of the AAO mask transferred on the Nb:$SrTiO_3$ substrate were shown in Figure S2. Pores with diameters of 80 nm (oxalic acid) and 230 nm (malonic acid), respectively, were arranged regularly. Due to the effect of phosphoric acid etching, these holes penetrate through to the substrate surface.

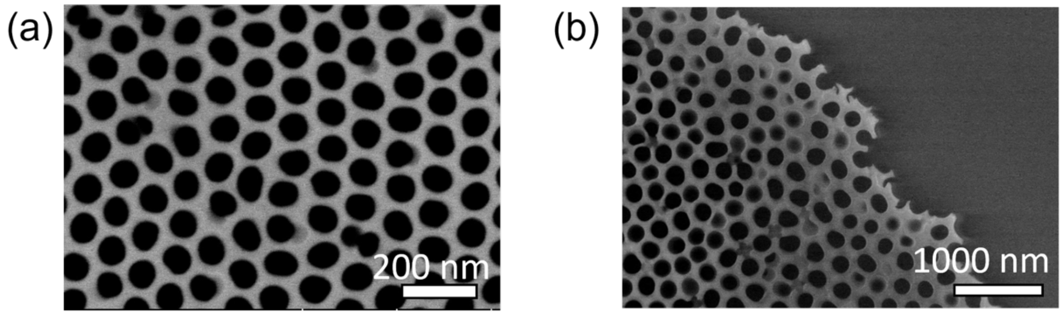

Figure S2. SEM image of (a) oxalic acid AAO and (b) malonic acid AAO on Nb: $SrTiO_3$ substrate.

Coverage of AAO mask and BFCO thin film

Since the AAO mask does not completely cover the Nb: $SrTiO_3$ substrate, the thin film, and nanodot areas will coexist after BFCO deposition, as seen in the SEM image. Therefore, the XRD signal included both the nanodot area and thin film area outside the AAO mask (Figure S3).

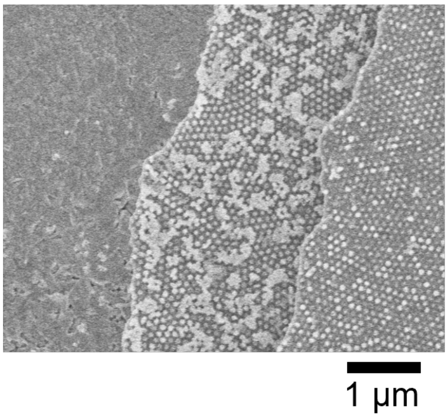

Figure S3. SEM image of deposited BFCO at the edge of the mask, after the AAO mask was removed. The thin film part (left side) and nanodot part (right side) coexist in the sample.

Time-dependent change of PFM image after poling

Figure S4 shows the out-of-plane PFM images of BFCO nanodot fabricated with oxalic acid AAO. These images are obtained 10 or 20 min. after the poling by a biased cantilever at −8 V. The purple contrast indicates the upward polarization resulting from the poling, which gradually disappears as the time after poling gets longer.

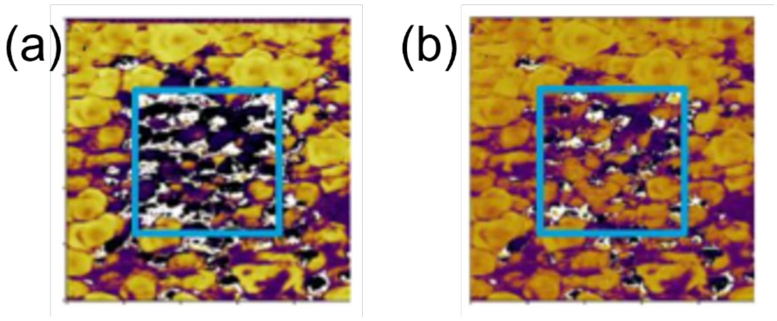

Figure S4. (a) Out-of-plane PFM images of BFCO oxalic acid AAO nanodot (a) 10 min after poling (b) 20 min after poling.

Reversal of MFM contrast by changing the magnetization direction of MFM tip

We have conducted MFM measurements with both upward and downward tip magnetizations. As seen in Figure S5, the domain geometry is unchanged but the contrast is reversed by the change of the tip magnetization. This confirms the MFM contrast truly reflects the magnetic signal.

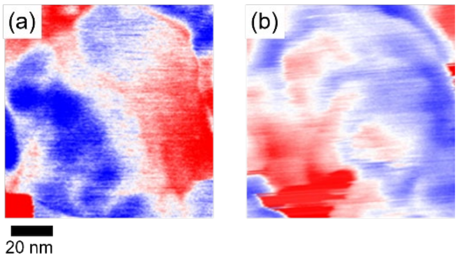

Figure S5. (a) MFM image of BFCO oxialic acid AAO nanodot (same figure as Figure 4(b)). (b) MFM image of same BFCO nanodot after magnetization direction on MFM tip was changed.
